# Genomic pneumococcal load and CSF cytokines are not related to outcome in Malawian adults with meningitis

**DOI:** 10.1016/j.jinf.2014.06.011

**Published:** 2014-11

**Authors:** Emma C. Wall, Jenna F. Gritzfeld, Matthew Scarborough, Katherine M.B. Ajdukiewicz, Mavuto Mukaka, Caroline Corless, David G. Lalloo, Stephen B. Gordon

**Affiliations:** a Malawi-Liverpool-Wellcome Trust Clinical Research Programme, Blantyre, Malawi; b Liverpool School of Tropical Medicine, Liverpool, UK; c College of Medicine, Department of Medicine, University of Malawi, Malawi; d John Radcliffe Hospital, Oxford, UK; e The University of Manchester Academic Health Science Centre, North Manchester General Hospital, UK; f Institute of Global Health, University of Liverpool, UK

**Keywords:** Bacterial meningitis, Streptococcus pneumoniae, Bacterial load, Cerebrospinal fluid, Cytokine response, Mortality, Africa

## Abstract

**Objective:**

Bacterial meningitis in sub-Saharan Africa is predominantly caused by *Streptococcus pneumoniae*, is often associated with HIV co-infection and mortality rates are double those seen in better resourced settings.

**Methods:**

To investigate the cause of this excessive mortality we quantified the pneumococcal DNA load and six common pro-inflammatory cytokines in the cerebrospinal fluid (CSF) of Malawian adults with culture proven pneumococcal meningitis and correlated the results to clinical parameters and outcome. There are currently no published data relating bacterial load to outcome in adults with pneumococcal meningitis.

**Results:**

The mean age of patients was 32 years, 82% were HIV infected and 49% had died by day 40. CSF bacterial loads were high (median 6.5 × 10^5^ copies/ml CSF) and there was no significant variation in bacterial load between survivors and non-survivors. All pro-inflammatory CSF cytokines were elevated in the CSF, with no clinically important differences between survivors and non-survivors. HIV status did not affect the CSF bacterial load or cytokine response.

**Conclusion:**

Mortality from pneumococcal meningitis in adults in sub-Saharan Africa is not related to pneumococcal bacterial load. More research is needed to understand the very high mortality from meningitis in this region.

## Highlights

• Pneumococcal meningitis in Malawian adults has a very high mortality rate.
• Bacterial load and cytokine response may predict outcome in children.
• Pneumococcal load and cytokine response were not associated with outcome in adults.
• Low CSF white cell count and Glasgow coma score were associated with mortality.
• Anti-cytokine therapies are unlikely to be effective in bacterial meningitis.

## Introduction

The incidence of pneumococcal meningitis in adults is estimated to be 0.1–1/100,000 in well-resourced countries^1^, ^2^; in sub-Saharan Africa where there are few surveillance data, the incidence is estimated to be 12/100,000 adult population^3^. In addition to the increased burden of disease in this region, the adult mortality rate from pneumococcal meningitis is 54%, compared to 30% in Europe.^4^, ^5^ High *Streptococcus pneumoniae* bacterial load in the cerebrospinal fluid (CSF) has been associated with increased mortality in children with meningitis in Malawi and Finland, higher bacterial loads in the blood of adults with pneumococcal sepsis in Europe are also associated with poor outcome.^6^, ^7^, ^8^ Pro-inflammatory cytokines are elevated in the CSF of adults with bacterial meningitis compared to viral meningitis^9^, ^10^ but little data are available to determine if elevated CSF cytokines are associated with outcome from meningitis in adults. We examined the association between pneumococcal load and host cytokine response and mortality from pneumococcal meningitis in Malawian adults.

## Methods

### Study participants

Patients aged >16 years with bacterial meningitis were recruited into one of two sequential randomised placebo controlled clinical trials of adjuvant therapy between 2001 and 2004 (dexamethasone, placebo) or 2006–2008 (glycerol, placebo)^11^, ^12^ conducted at Queen Elizabeth Central Hospital, Malawi. CSF samples taken prior to antibiotic and adjuvant therapy were transported immediately to the laboratory where a cell count was performed. If the cell count met the inclusion criteria for the contributing clinical trial (>100 cells/mm^3^ with >50% neutrophils),^11^, ^12^ CSF supernatant was frozen at −80 °C within 2 h of lumbar puncture. The laboratory at the Malawi-Liverpool-Wellcome Trust clinical research programme has provided an externally quality-controlled microbiology service to QECH since 2000 www.mlw.medcol.mw. Diagnostic CSF was cultured on blood agar at 37 °C in 5% CO_2_. *S. pneumoniae* was identified by standard methods including optochin sensitivity and alpha haemolysis. Only culture positive samples for *S. pneumoniae* were included in this study, molecular diagnostics using PCR were not available. Treatment of pneumococcal meningitis was ceftriaxone 2 g twice daily for 10 days. A second CSF sample was taken 48 h post antibiotic therapy in a sub-set of patients, some of whom were treated with intramuscular as opposed to intravenous ceftriaxone as per protocol of the dexamethasone clinical trial. Poor outcome was defined as death by 6 weeks of follow up. Morbidity data were not available.

### Real time PCR for *S. pneumoniae*

DNA was extracted from 200 μl of pneumococcal culture positive CSF supernatant and Real-Time PCR was performed as described previously using autolysin (*LytA*) as the amplification target.^13^ Standard curves were created using purified genomic DNA extracted from *S. pneumoniae* serotype 23F (P833), and quantified using the NanoDrop ND-1000.

### Cytokine analysis

Six cytokines IL1β, IL6, IL10, IL8, IL12 and TNFα were measured using a cytometric bead array (BD Biosciences, San Diego). Six bead populations with differing 650 nm fluorescence intensities were coated with cytokine specific capture antibodies and incubated with flourochrome (phycoerythrin – 585 nm) according to the manufacturer's instructions. The resulting sandwich complexes were resolved in the FACScan flow cytometer and the output analysed using manufacturer's software.

### Ethics

Participants or accompanying legal guardians gave written informed consent for CSF samples to be stored and used for research studies. Both constituent studies were granted ethical approval for sample collection and analysis by the Liverpool School of Tropical Medicine Research Ethics Committee and the College of Medicine Research Ethics Committee, University of Malawi.

### Statistics

Statistical analysis was done using IBM SPSS statistics version 20. Graphs were generated using GraphPad PRISM version 5. Median (inter-quartile range) was used to summarise non-normally distributed variables, while mean (SD) was used to summarise normally distributed variables. Statistical tests were 2 tailed, non-parametric tests were used to analyse data that were not normally distributed. A *t*-test was used to analyse normally distributed variables. Categorical variables were analysed using Fisher's exact test. A multivariate model was generated using binary logistic regression, enter mode, and cross checked using backwards LR mode. Clinical variables entered into the multivariate model were chosen based on previous association with poor outcome.^4^ Statistical significance was determined at a value of <0.05 and 95% confidence intervals for the odds ratios have been provided. Day 40 was used as the outcome measure for all analyses.

## Results

Data were collected from 151 patients with stored CSF samples and paired clinical data. Data were available for all patients to day 10; day 40 outcome data were missing for 3 patients who were lost to clinical follow up.

**Figure 1:**
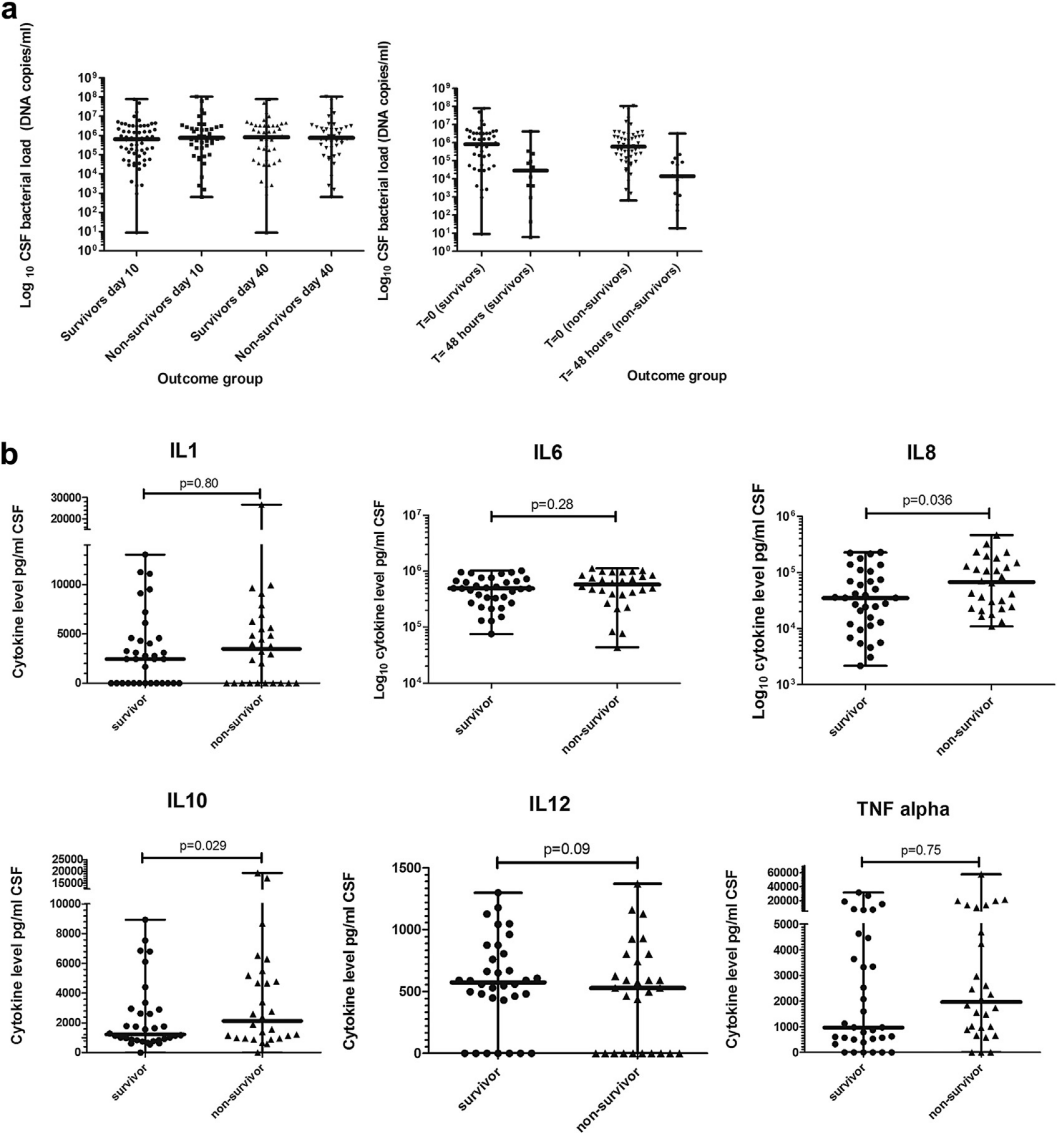
CSF parameters in adults with pneumococcal meningitis in Malawi. Bars indicate median with range. 1a log 10 CSF bacterial load in survivors and non-survivors at 10 and 40 days post admission to hospital and CSF bacterial load in day 40 survivors and non survivors at admission and 48 h post antibiotic treatment. 1b CSF cytokine levels in survivors and non-survivors at day 40 paired by cytokine measured with full range.

Baseline characteristics of the included patients are shown in Table 1. The mean age was 32 years (IQR 25–36); 51% were female and 82% were HIV-antibody positive of which only 2 were on Antiretroviral therapy (ART) (Table 1). The overall mortality was 63/151 (41%) at day 10 and 73/148 (49%) at day 40. Data on sequelae in survivors were not available for analysis. Median CSF white cell count (WCC) was 760 cells/mm^3^ (IQR 181–2600) with significantly higher WCCs in survivors compared to non-survivors *p* = 0.02 on univariate analysis (Table 1). The median CSF bacterial load was 6.5 × 10^5^ copies/ml (IQR 1.08 × 10^5^–2.96 × 10^6^) in the admission samples and 2.96 × 10^4^ copies/ml (IQR 3.8 × 10^3^–2.12 × 10^5^) in the CSF samples taken 48 h post antibiotics (Fig. 1a). There was no difference in the bacterial load between survivors or non-survivors at presentation or at 48 h (*p* = 0.52 and 0.65 respectively, Table 1). In addition there was no significant difference in the magnitude of the decline in the bacterial load between survivors and non-survivors over 48 h. An ROC curve was synthesised to assess if bacterial loads >1 × 10^6^ copies/ml predicted poor outcome; the area under the curve (AUC) was 0.49 (non-significant, curve not shown).

**Table 1:** Baseline characteristics for patients with culture-confirmed pneumococcal meningitis and CSF bacterial load values (*n* = 102). Multivariate analysis presented for Model 1, clinical parameters with bacterial load data.

Clinical parameter (*n* = data available)	All participants (SD, IQR or %)	Survivor day 40 (SD, IQR or %)*n* = 75	Non-survivor day 40 (SD, IQR or %)*n* = 73	Survival significance (univariate)	Odds ratio (95% CI) bacterial load data Model 1	Survival significance bacterial load model 1 *n* = 102 (multivariate)
Female gender *n* = 144	72 (51%)	36 (48%)	36 (49%)	0.4^c^	2.24 (0.62:8.03)	0.21
Mean age *n* = 144	32.1 (SD 9.7)	30 (SD 9.1)	33 (SD 10.2)	0.06^a^	1.02 (0.95:1.09)	0.54
HIV antibody positive *n* = 141	115 (82%)	60 (80%)	55 (75%)	0.8^c^	1.43 (0.25:8.2)	0.68
Seizures *n* = 142	50 (35%)	16 (21%)	34 (47%)	0.002^c^	7.2 (2.18:23.8)	0.001
Altered mental state GCS <15	111 (81%)	49 (65%)	61 (84%)	<0.001^c^	4.55 (0.41:51.0)	0.22
Median Glasgow coma score (GCS) *n* = 137	11 (8–14)	13 (10–15)	9 (7–11)	<0.001^b^	0.72 (0.59:0.92)	0.001
Dexamethasone *n* = 89	49/89 (55%)	28 (57%)	21 (43%)	0.36^c^	0.47 (0.12:1.87)	0.28
Glycerol *n* = 62	27/62 (46%)	8 (30%)	19 (70%)	0.03^c^	2.29 (0.4:12.9)	0.34
Mean Haemoglobin (g/dL) *n* = 129	11.3 (SD 2.9)	11.3 (SD 2.4)	11.4 (SD 3.2)	0.94^a^	0.82 (0.68:0.98)	0.032
Median CSF white cell count (mm^3^/ml) *n* = 143	760 (181–2600)	1120 (320–1320)	370 (105–1480)	0.021^b^	1.0 (1.0–1.0)	0.91
**Median CSF bacterial load** **(DNA copies/ml) with IQR**						
Admission *n* = 102	6.5 × 10^5^ (1.08x10^5^–2.96 × 10^6^)	8.10 × 10^5^ (4.74 × 10^4^–3.24 × 10^6^)	5.94 × 10^5^ (1.59 × 10^5^–2.42 × 10^6^)	0.52^b^	1.0 (1.0–1.0)	0.64
48 h post antibiotics *n* = 42	2.9 × 10^4^ (3.8x10^3^–2.12 × 10^5^)	2.72 × 10^4^ (4.12 × 10^3^–2.43 × 10^5^)	7.96 × 10^4^ (1.56 × 10^3^–2.19 × 10^5^)	0.64^b^	1.0 (1.0–1.0)	0.22

a *t*-test.

b Mann–Whitney-*U* test.

c Fisher exact test/Chi-squared test.

Six common cytokines were measured in the CSF. Overall there was an intense pro-inflammatory cytokine response in the CSF of all patients; no differences by day 40 outcome reached statistical significance on univariate analysis (Supplementary Table 1, Fig. 1b). Two multivariate models were synthesised to investigate the influence on outcome of bacterial load (model 1) and cytokine response (model 2). Two models were required as there were insufficient patients with both bacterial load and cytokine data (*n* = 18) to combine into a single model. No statistically significant mortality association was demonstrated for the CSF bacterial load or CSF white cell count, HIV status, age or gender on model 1 (*n* = 102); seizures at any time in the illness, GCS or altered mental status and anaemia were associated with mortality (Table 1). In model 2 (*n* = 62) IL8 and IL10 were marginal predictors of non-survival; IL8 *p* = 0.036, OR 1.00 (95% CI 1.00: 1.00) and IL10 *p* = 0.029, OR 1.00 (95% CI 1.00 : 1.00); of the clinical parameters, only altered mental status or GCS retained significance in this model (Supplementary Table 1).

## Discussion

We have previously shown that coma, seizures and anaemia predict poor outcome from bacterial meningitis in Malawi,^5^ but the causes of the excess mortality compared to patients in more well-resourced settings remain unclear. In this study, there was no difference in the bacterial load and only marginal difference in the cytokine response between survivors and non-survivors despite lower CSF white cell counts in non-survivors. Our findings are markedly different to data in children with pneumococcal meningitis in Malawi and Europe, and adults with pneumococcal bacteraemia in Europe or meningococcal meningitis in the UK.^6^, ^7^, ^8^, ^14^ No published data has quantified CSF pneumococcal load in adults with meningitis in either setting. The lack of association between outcome and pneumococcal load, in contrast to these other studies was unexpected. HIV uninfected adults with pneumococcal meningitis in Europe have a 10 fold higher CSF WCC than our patients, a CSF WCC of <1000 cells/mm^3^ has been shown to be significantly associated with mortality in Europe.^4^ In our study, the median CSF WCC was substantially below this threshold, and low CSF WCCs were associated with poor outcome. We hypothesise that in adults with pneumococcal meningitis in Malawi, rapid bacterial growth occurs within the CSF with relatively little restriction by the host immune response, leading to high bacterial loads in both outcome groups. In addition, delays from symptom onset to admission in the community and to lumbar puncture within the hospital system may have resulted in the bacterial growth reaching the plateau, as opposed to the exponential growth phase in the CSF by the time of lumbar puncture, and hence any differences between outcome groups may have equalised by the time of examination. Time from symptom onset to lumbar puncture in the included studies was 3–5 days, compared to <48–72 h for most European studies.^11^, ^12^, ^15^, ^16^, ^17^

Adults with pneumococcal meningitis in Malawi have different baseline characteristics compared to those studied in other settings outside of sub-Saharan Africa,^4^, ^5^ and disease is caused disproportionately by serotype one.^18^ Data from studies of pneumococcal meningitis in this region may not be directly comparable to data from other regions. Adults with bacterial meningitis in Malawi are commonly HIV co-infected, they present late to hospital and they experience in-hospital delays to lumbar puncture and antibiotic treatment.^5^, ^19^ 82% of the patients in our study were HIV-antibody positive compared to 62% of children with pneumococcal meningitis in Malawi and 0% from the European series.^6^, ^7^ The CSF levels of six common cytokines in our patients were much higher than those observed at baseline in predominantly HIV-uninfected adults with bacterial meningitis in other centres,^9^, ^20^, ^21^ although we cannot exclude the possibility that a pro-inflammatory effect of untreated HIV infection is contributing to the cytokine reaction observed, as the numbers of HIV un-infected patients in our study were small.^17^ Minimal data exist correlating cytokine levels with poor outcome in a small number of patients with meningitis, no HIV co-infected patients were included in that cohort.^9^ CSF cytokine levels in our study did not vary by adjunct or placebo in either trial, dexamethasone had no effect on outcome.^11^

The paediatric study in our centre, where 62% of children where HIV co-infected, demonstrated an equally intense CSF cytokine response in children with pneumococcal meningitis; the four cytokines measured in that study (TNFα, IL-1β, IL-6 and IL-10) were significantly higher only in HIV co-infected non-survivors as compared to HIV co-infected survivors.^5^ HIV status is not a predictor of poor outcome in adults with pneumococcal meningitis in Malawi,^4^ although it is well established that HIV infection is an important risk factor for invasive pneumococcal disease (IPD) in sub-Saharan Africa.^18^

We have previously reported results of a major proteomic analysis of Malawian adults with bacterial meningitis.^22^ Although increased CSF protein spots were associated with non-survival, no differences in the host proteomic response other than complement and ferritin responses were noted. In addition we have observed that the persistence of pneumolysin in the presence of a falling bacterial load and low CSF complement C3 were associated with higher mortality in a small number of patients with pneumococcal meningitis.^12^, ^15^ Those data, combined with the observations in this study of lower WCC and higher cytokines in the CSF, suggest that poor outcome may be due more to abnormalities of the host response to *S. pneumoniae* than excessive virulence of the pathogen.^13^, ^23^ CSF co-infection with Epstein–Barr virus has also been shown to correlate with poor outcome in adults with bacterial meningitis in Malawi.^24^ We are currently investigating whether lower CSF WCCs are associated with viral co-infection. In addition, the influence of significant pre-hospital and clinical delays on outcome has not been fully quantified.^5^, ^13^

Limitations exist within our data. Firstly, the small numbers of patients with both CSF genomic load and cytokine data available precluded a definitive analysis of bacterial load and cytokine levels in the same statistical model. Secondly, data for sequelae in survivors were not available, the different scoring systems used in the included studies meant significant heterogeneity in the outcome data precluded analysis of the impact of bacterial load or cytokine response on morbidity from meningitis.

In conclusion, poor outcome from pneumococcal meningitis in Malawi is likely to be multifactorial and our data suggest that anti-cytokine adjunctive treatments in sub-Saharan Africa are unlikely to be effective. Alternative strategies such as pneumococcal vaccination in HIV infected adults, reducing pre-hospital delays to treatment, optimising in-hospital care, investigating alternative adjunctive treatments targeting pneumococcal toxins and optimising macrophage phagocytosis^13^, ^23^, ^25^, ^26^, ^27^, should be on-going research priorities.

## Funding

The bacterial load work was funded by the 10.13039/100004440Wellcome Trust (CDF 061231 and 089671/B/09/Z) (Clinical PhD fellowship to EW) and NIHR Biomedical Research funding to SG. The cytokine analysis was funded by the Wellcome Trust (Research fellowship to SBG). The steroid and glycerol adjunctive therapy studies were funded by the 10.13039/501100000403Meningitis Research Foundation. Neither the funding bodies nor the trial sponsors had any role in the laboratory work, data analysis, manuscript preparation or decision to publish.

## Conflict of interest

The authors declare no conflicts of interest.
