# Supplementary material for: Genomic pneumococcal load and CSF cytokines are not related to outcome in Malawian adults with meningitis
Source: J Infect. 2014 Nov;69(5):440–6. doi: 10.1016/j.jinf.2014.06.011 (PMC4209731; doi:10.1016/j.jinf.2014.06.011)
Supplement: Supplementary file 1 [file mmc1.docx]

| **Clinical parameter (n=data available)** | **All participants (SD, IQR or %)** | **Survivor**  **day 40**  **(SD, IQR or %)**  **n=34** | **Non-survivor**  **day 40**  **(SD, IQR or %)**  **n=28** | **Survival significance (univariate)** | **Odds ratio (95% CI)**  **Cytokine data**  **Model 2** | **Survival significance Cytokines Model 2** |
| --- | --- | --- | --- | --- | --- | --- |
| Female gender  n=62 | 26 (42%) | 17 (50%) | 9 (32%) | 0.20 # | 1.08  (0.16 : 7.04) | 0.93 |
| Mean age  n=62 | 30.8 (SD 9.2) | 29 (SD 9.1) | 32 (SD 9.3) | 0.19 † | 1.08  (0.98 : 1.19) | 0.10 |
| HIV antibody positive n=60 | 51 (85%) | 27 (79%) | 24 (86%) | 0.42 # | 0.41  (0.02 : 7.2) | 0.54 |
| Seizures  n= 62 | 14 (23%) | 8 (24%) | 6 (21%) | 0.54 # | 0.18  (0.02 : 1.4) | 0.10 |
| Altered mental state GCS <15 n=62 | 53 (85%) | 26 (76%) | 27 (96%) | 0.033 # | 19.23  0.89 : 417) | 0.06 |
| Median Glasgow coma score (GCS) n=62 | 10 (8-13) | 11 (9-14) | 9 (7-10) | 0.004 ‡ | 0.73  (0.55 : 0.99) | 0.005 |
| Dexamethasone | 32 (52%) | 17 (50%) | 15 (54%) | 0.80 # | 0.49  (0.08 : 2.9) | 0.43 |
| Mean Haemoglobin (g/dL) n= 57 | 11.5 (SD 2.6) | 11.1 (SD 2.4) | 11.9 (SD 2.8) | 0.52 † | 1.09  (0.76 : 1.5) | 0.61 |
| Median CSF white cell count (mm^3^/ml) n=62 | 1142  (296 – 2880) | 1360  (437 – 4280) | 720  (198 – 2160) | 0.13 ‡ | 1.0  (1.0 : 1.0) | 0.19 |
| **Median CSF cytokine values in pg/ml with IQR n=67** |  |  |  |  |  |  |
| TNF alpha | 1575  (576-4644) | 973  (474-4499) | 1966  (908-8636) | 0.44 ‡ | 1.0  (1.0 : 1.0) | 0.75 |
| IL-1 | 2779  (0-5511) | 2450  (0-4558) | 3482  (0-6085) | 0.31 ‡ | 1.0  (1.0 : 1.0) | 0.80 |
| IL-6 | 497974  (335090–764513) | 492416  (271718-688485) | 576186  (382122-842062) | 0.62 ‡ | 1.0  (1.0 : 1.0) | 0.28 |
| IL-8 | 40334  (19961–115057) | 35216  (11818-81961) | 67513  (23386-143534) | 0.20 ‡ | 1.000009  (1.000001 : 1.000017) | 0.036 |
| IL-10 | 1568  (909-4475) | 1233  (832–2966) | 2130  (959-5086) | 0.44 ‡ | 1.00028  (1.000028 : 1.00054) | 0.029 |
| IL-12 | 560  (0-804) | 547  (443-822) | 528  (0-729) | 0.98 ‡ | 0.99  (0.996 : 1.000018) | 0.09 |

†= t-test,‡ = Mann-whitney-U test, # = Fisher exact test/Chi squared test

**Legend for supplementary Table 1: Baseline characteristics and CSF cytokine values for patients with culture-confirmed pneumococcal meningitis.**
